# Democratization of Point-of-Care Viral Biosensors: Bridging the Gap from Academia to the Clinic

**DOI:** 10.3390/bios15070436

**Published:** 2025-07-07

**Authors:** Westley Van Zant, Partha Ray

**Affiliations:** Department of Medicine, Division of Infectious Diseases and Global Public Health, University of California, San Diego, CA 92093, USA

**Keywords:** biosensors, accessible diagnostics, point-of-care (POC) testing, democratization

## Abstract

The COVID-19 pandemic and recent viral outbreaks have highlighted the need for viral diagnostics that balance accuracy with accessibility. While traditional laboratory methods remain essential, point-of-care solutions are critical for decentralized testing at the population level. However, a gap persists between academic proof-of-concept studies and clinically viable tools, with novel technologies remaining inaccessible to clinics due to cost, complexity, training, and logistical constraints. Recent advances in surface functionalization, assay simplification, multiplexing, and performance in complex media have improved the feasibility of both optical and non-optical sensing techniques. These innovations, coupled with scalable manufacturing methods such as 3D printing and streamlined hardware production, pave the way for practical deployment in real-world settings. Additionally, software-assisted data interpretation, through simplified readouts, smartphone integration, and machine learning, enables the broader use of diagnostics once limited to experts. This review explores improvements in viral diagnostic approaches, including colorimetric, optical, and electrochemical assays, showcasing their potential for democratization efforts targeting the clinic. We also examine trends such as open-source hardware, modular assay design, and standardized reporting, which collectively reduce barriers to clinical adoption and the public dissemination of information. By analyzing these interdisciplinary advances, we demonstrate how emerging technologies can mature into accessible, low-cost diagnostic tools for widespread testing.

## 1. Introduction

In recent years, viral diagnostic technologies have evolved significantly, rapidly improving to meet the demand for quick, accurate, and accessible testing capabilities. Given the emergence of large-scale viral infections such as avian influenza (H5N1) [1], Ebola [2], and the Zika virus [3], the need for advancements in diagnostic technologies remains pressing. Effectively diagnosing emerging viruses, especially in the early stages of infection, is critical to prevent localized outbreaks from reaching a global scale [4]. The ongoing COVID-19 pandemic, caused by SARS-CoV-2, illustrates the severe consequences of failing to control localized endemic events before the pathogen spreads through global markets and travel systems. The pandemic also highlighted the demand for effective diagnostics to extend beyond the laboratory, with population-level testing pushing diagnostics toward at-home and point-of-care (POC) settings [5]. This has pushed development efforts to expand in several directions, with enhancements in sensitivity and accuracy balanced with cost reduction, miniaturization, scalability for mass production, and simplified sampling and data processing [6]. The World Health Organization (WHO) broadly characterizes ideal POC diagnostics according to their real-time connectivity, ease of specimen collection, affordability, sensitivity, specificity, user-friendliness, rapidness, and robustness [7]. Advancements in these characteristics address the challenges posed by emerging and ongoing infectious diseases and democratize access to diagnostics, both in their administration and in interpreting their results.

**Figure 1:**
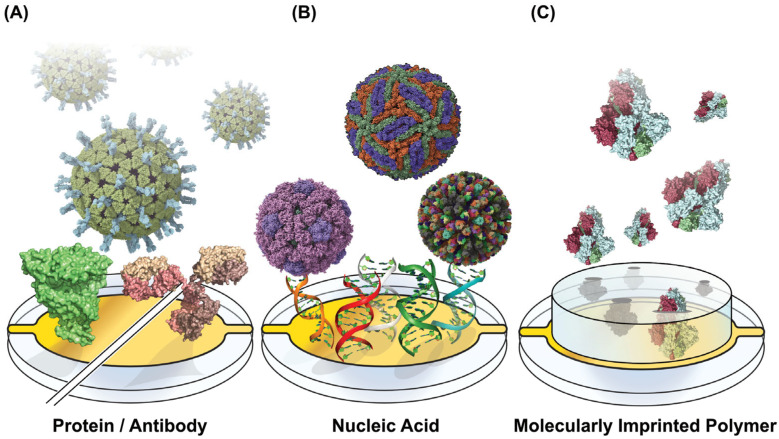
Capture Motif-Functionalized Biosensor Surfaces. Sensing surfaces are functionalized across the various viral diagnostic approaches with motifs for capturing proteins and whole virions. (**A**) Proteins can be surface-tethered capture moieties, with virus-binding proteins and antibodies providing specific binding (PDB IDs 4V7Q, 1R42, 1IGT). (**B**) Surface-linked nucleic acids can serve a similar viral capture function while providing additional stability and cost reduction. Their programmability enables multiplexed detection for sensing multiple analytes on one sensor surface (PDB IDs 5IRE, 1CD3, 3KZ4). (**C**) Molecularly imprinted polymers (MIPS) can be pre-produced to capture viruses with cavities imprinted to capture specific viral proteins (PDB ID 6VXX). Structures were rendered using UCSF ChimeraX 1.9 [39], and figures were generated using Adobe Illustrator version 24.

At its core, a biosensor typically includes a biorecognition element designed to capture diagnostic targets selectively. These targets include nucleic acids, viral proteins, antibodies, and entire virions [8,9]. The capture elements for these biomarkers can take various forms, such as antibodies (both whole and functional fragments) and proteins (Figure 1A), or nucleic acids (Figure 1B), like DNA, RNA, and aptamers [10]. Alternatively, artificial receptors like molecularly imprinted polymers (MIPs) can be templated with viral proteins as a synthetic alternative to protein or nucleic acid capture [11] (Figure 1C). Combining multiple biorecognition elements also enables the simultaneous detection of multiple analytes, including multiple viruses or antigens from one pathogen [12]. Accompanying the biorecognition element is a form of signal transduction that translates the capture of the biomarker into a measurable readout [13]. Optical signaling methods rely on changes in light properties, including refractive index, fluorescence, color changes, absorbance, or light interference [14]. Electrochemical signal transduction can appear in several forms, including amperometric readouts that measure current, potentiometric readouts of voltage, and changes in impedance [15]. Biosensors can also integrate mechanical signal transduction, exemplified by quartz crystal microbalance (QCM) and microcantilever sensors [16,17].

Furthermore, signal transduction may involve thermal or magnetic methods [18,19]. Signal amplification techniques can be employed to lower detection limits and enhance the readout of weak signals. Nanomaterials such as colloidal gold [20], quantum dots [21], and graphene nanotubes [22] can be combined with biorecognition elements to improve the quality and strength of faint signals. While some diagnostics like lateral flow assays (LFAs) provide a simple binary readout that users can interpret directly [23], other diagnostic methods require post-processing to convert the signal into a comprehensible output [24]. Various post-processing methods are employed, from software interfaces and smartphone apps that interpret results to more complex machine learning (ML) models designed to handle increasingly intricate datasets [25,26]. Moreover, viral diagnostics often utilize microfluidic sample delivery systems, which assist users by reducing sample volumes, directing the specimen to the sensing region, and minimizing the risk of human error associated with manual sample processing [27,28].

Multiple approaches to viral diagnostics can be taken, depending on the nature of the viral recognition target. The detection of viral genomes has become particularly ubiquitous due to the COVID-19 pandemic, and methods such as real-time reverse transcription polymerase chain reaction (RT-PCR), next-generation sequencing (NGS), and reverse-transcription loop-mediated isothermal amplification (RT-LAMP) have all undergone significant development in recent years [29,30]. While RT-PCR serves as the gold standard for viral detection due to its rapid time-to-result, high specificity, and low copy number requirements, it requires specialized instrumentation, trained personnel, and the production and storage of reagents such as polymerases and primers [31]. RT-LAMP does improve accessibility by eliminating the need for a thermal cycler, but it has also seen limited adoption in the clinic [32]. Other diagnostic methods target viral proteins or antibodies raised against them rather than genomic information. Methods like ELISA are the standard for protein-based diagnostics, but they also suffer from limitations in personnel, sample handling, and costly reagents like antibodies [33]. In this sense, there is a tradeoff in the diagnostic methods already demonstrated in point-of-care settings, where higher sensitivity often accompanies complex assay administration, expensive reagents, and a higher barrier for understanding assay readouts.

Across the scientific community, there is no shortage of promising publications effectively demonstrating advancements in viral detection from the standpoint of instrumentation and methodology. However, barriers still exist in bringing these new advancements to fruition in the clinic itself. In addition to logistical constraints like manufacturing costs and scalability, an underlying cause is the current prioritization dominating the research world. Development efforts centered around high-complexity instrumentation based on well-established local infrastructure can exclude disenfranchised communities from accessing novel diagnostic technologies [34]. Further contributing to this may be the cyclical nature of research funding, where pursuing novel assay developments rather than focusing on maturing established technologies can present a far more intellectually and financially viable approach when compared to addressing needs for cost improvements, multiplexing, and time-to-results. On the commercial side of research and development, diagnostic products are also frequently neglected because the contagious diseases prevalent in low-income countries do not yield substantial economic returns for biotech companies—this lack of profitability results in insufficient investment in their development [35]. Until this mindset largely shifts, this cycle of seeking new research areas rather than honing existing methodologies will likely continue.

Factors like the need to control local outbreaks, expand accessibility to disenfranchised communities, and conduct population-scale testing mean diagnostics must expand beyond central laboratories, regardless of how technically effective lab-oriented methods may be. One potential solution to this issue is establishing partnerships between academic institutions, industry, regulatory agencies, and government. In this model, the most promising diagnostic products developed in academic laboratories would receive targeted financial incentives from the government and be manufactured by the industrial partners with approval from the regulators. This collaboration could significantly advance the development of vital diagnostic products for infectious diseases. The feasibility of this approach has been demonstrated in the recent NIH Rapid Acceleration of Diagnostics (RADx) initiative, which provides companies with small business innovation research (SBIR) and small business technology transfer (STTR) grants to aid in the rapid development of COVID-19 diagnostics alongside universities and regulatory bodies [36,37]. Expanding such programs beyond COVID-19 could help address the diagnostic gaps for other infectious diseases, leveraging this new but now-proven collaborative model.

We must recognize the urgent need for solutions to infectious diseases. Access to effective diagnostics and treatments is crucial for the health and well-being of millions. By prioritizing this partnership, we can drive meaningful progress and tackle the critical health challenges that underserved populations face. In today’s interconnected world, no geographical barrier can prevent infectious diseases from crossing borders. The rapid globalization of trade and travel has facilitated the movement of people and goods around the globe, increasing the likelihood of pathogens being transmitted from one location to another [38]. In this environment, outbreaks can escalate quickly, as seen in the case of diseases like COVID-19, which spread swiftly from one country to another. This highlights the vulnerabilities of public health systems worldwide.

In this review, we discuss a variety of advancements in viral diagnostics that help bring assays developed in academic labs closer to the clinic for real-world implementation. We highlight that across the several approaches to viral biosensing, from optical to non-optical methods, improvements in cost, sensor design, and assay administration have greatly improved the accessibility and breadth of novel diagnostics. While many of the advancements discussed have not yet been implemented in POC settings, they demonstrate the ever-moving push to bridge the gap between POC accessibility and laboratory diagnostic technology. Further, we discuss the production processes that improve the feasibility of pushing state-of-the-art diagnostics into clinical settings by lowering costs, enabling local component manufacturing, and simplifying both diagnostic administration and reporting through pre-functionalized sensors and out-of-the-box software tools. Finally, we discuss accessibility improvements in scientific reporting, which support the transition of novel diagnostics into the clinic through administrative shifts towards accessible and understandable literature and reporting.

## 2. Optical Methods

### 2.1. Surface Plasmon Resonance

**Figure 2:**
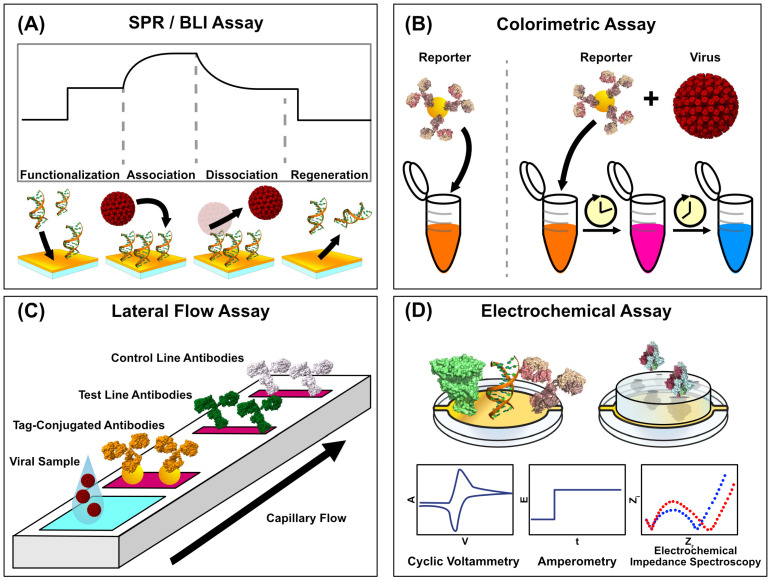
Schematics of Viral Biosensing Approaches. Viral biosensing can be achieved in a variety of ways. (**A**) Label-free biosensing methods can track the association of surface-immobilized capture motifs and analytes in real time, giving rise to kinetic and binding affinity parameters. (**B**) Colorimetric assays detect viral particles through the association of viral particles with reporter-conjugated capture moieties, producing a distinct visible readout with simple assay administration. Viral detection is coupled with a progressive, visible color change in the sample, while a virus-free sample remains as the same starting color. (**C**) Lateral flow assays are a clinical standard due to their relative simplicity and binary readout resulting from reporter-conjugated binding activity coupled with viral binding. The different antibodies, consisting of control, test, and tag-conjugated, are colored in white, green, and orange respectively to illustrate the multiple regions through which the sample flows. (**D**) Electrochemical assays can utilize the electrical signals associated with chemical binding events, and they can take several approaches, which include cyclic voltammetry, amperometry, and electrochemical impedance spectroscopy. (PDB IDs: 1D28, 4V7Q, 1IGT, 1R42, 6VXX). Structures were rendered in UCSF ChimeraX 1.9 [39], and figures were generated in Affinity Designer 2.

Surface Plasmon Resonance (SPR) is a label-free optical technique that utilizes photon–electron interactions with plasmonic materials. The method operates through the phenomenon of light under ideal matching conditions, undergoing total internal reflection at the interface of a thin metal film and a dielectric. Tracking the angle of light at which the total internal reflection occurs enables the real-time analysis of binding events since changes in the refractive index will shift the angle of incidence [40] (Figure 2A). SPR biosensing is commonplace in pharmaceutical research due to its ability to study binding affinities between biomolecules. It is well-suited to screening drugs at on and off-rates and affinities. Still, it can also detect the presence of antibodies and viral antigens. The absence of labels means that the interactions being studied are closer to their native interactive states, and the method is exceptionally sensitive [41]. The technique is not without flaws, however. Typical commercial instrumentation is often expensive, and costs per run are high due to the use of plasmonic metals (like gold and silver) in chip production. Still, instruments frequently costing hundreds of thousands of dollars cannot be feasibly implemented in clinics. Further, while SPR is straightforward to train, it requires the additional considerations of buffer preparation and system design to minimize bulk refractive index shifts and non-specific surface binding [42], which makes it adoption in clinics difficult. Further, multiplexed sensing is limited by the number of fluidic channels, which is a constraint that proves difficult to overcome with expensive and closed-source instrumentation.

Viral SPR biosensing is by no means a novel technique, however. For instance, Riedel et al. demonstrated an SPR biosensor for detecting the Epstein–Barr virus in patient sera in 2014 [43]. Similarly, Florschütz et al. used surface-immobilized nucleotides to capture the barley stripe mosaic virus RNA (BSMV) in 2013 [44]. More recently, Yano et al. demonstrated a SARS-CoV-2 SPR biosensor that detected the nucleocapsid, claiming a LOD as low as 85 fM, in which surface-immobilized anti-nucleocapsid antibodies were used for capture [45]. In contrast, secondary antibody-conjugated AuNPs were used for amplification. Surface-immobilized aptamers have also been used for viral detection, with sensors for the HIV-1 Tat protein [46] and the SARS-CoV-2 N-protein in complex media [47], which have been shown using commercial instrumentation.

While these studies demonstrate the method’s ability to detect viral analytes, recent work has aimed to push SPR closer to clinical relevance. Recently, a portable SPR system for analyzing nasopharyngeal COVID-19 tests has been demonstrated, using covalently linked, RBD-binding nanobodies on the chip surface. The study utilized a small-footprint SPR spectrometer [48], which is a fraction of the size of other commercial SPR instruments, and machine learning was incorporated to help identify positive signal shifts. In another SARS-CoV-2 assay, a four-channel SPR chip was immobilized with the spike protein and the human ACE2 receptor to detect antibodies and viral particles in serum samples [49].

The size reduction of SPR instruments is gradually progressing, with instruments from the likes of OpenSPR (Nicoya, Kitchener, ON, Canada) [50], NanoSPR (NanoSPR, Chicago, IL, USA) [51], and Plasmetrix (Plasmetrix, Montreal, QC, Canada) [52] demonstrating how SPR instruments can easily find a place in the clinic where larger commercial instruments could not. In addition to their physical footprint, sensor surface advancements have also been made to improve the clinical adoptability of SPR. Wallace et al. showed the development of disposable, multiplexed plasmonic chip surfaces with multiple discrete sensing regions that could detect SARS-CoV-2, Norovirus, and Zika [12]. The pre-functionalized surface allowed them to be produced and shipped to clinics, ready to use out of the box; this is alongside improvements in the size of recent instrumentation. While this system was centered around hyperspectral polarimetry, the plasmonic nature of the chip design means it could be implemented in SPR systems, and more importantly, pre-functionalized surfaces reduce the complexity of running assays in a clinic. While SPR systems are already sufficiently sensitive to detect viral biomarkers in patient samples, there is still work regarding miniaturization, adaptable chip surfaces, and multiplexed sensing methodologies to push them closer to clinical adoption. There is an unavoidable cost regarding sensor chips due to their reliance on rare metals, but reusable surfaces and multiplexed sensing can help achieve more diagnoses per chip investment.

### 2.2. Biolayer Interferometry

Biolayer interferometry is gaining traction as another versatile tool for detecting viral infections. BLI systems characterize binding interactions through a recognition ligand coupled to a sensor tip. Interactions with analytes in solution occur at the tip, creating a thin layer that causes interference in light passing through the length of the sensor [53]. Light bouncing off a ligand-binding and a stationary reference surface causes an interference pattern, which induces a measurable wavelength shift. Since the wavelength shift is proportional to the thickness of the layer, active measurement enables real-time binding analysis [54], which can elucidate kinetic parameters as biomarkers bind. Measuring the biomolecular interactions of analytes directly in solution and minimizing sample workup procedures can make it well-suited to point-of-care applications. Like SPR, biolayer interferometry carries the benefits of sensitive, real-time, and label-free biosensing (Figure 2A). Still, careful surface design and considerations like non-specific binding and surface regeneration are required. BLI avoids the time-consuming steps of washing and visualization necessary for ELISA diagnostics, making it more consistent in the hands of clinicians. BLI has been used to develop viral inhibitors, such as therapeutic antibody epitope mapping [55] and the characterization of inhibitors for viral infections [56,57]. Still, its applicability extends into diagnostics as well. In addition to simplified sensor preparation, BLI’s applicability in the clinic can be further bolstered with more robust capture probes on the sensor surface. Poolsup et al. demonstrated a BLI aptasensor to detect SARS-CoV-2 anti-nucleocapsid antibodies in human saliva [58]. By using aptamers rather than protein-based capture ligands, the sensor can be made far more robust, which is especially conducive for sensors that will be mass-manufactured and distributed to clinics [9].

Numerous studies have utilized BLI to characterize virus–ligand interactions, and several have tried to democratize novel viral diagnostics for clinical use. For instance, Dzimianski et al. demonstrated a single-use BLI assay for detecting antibodies against the SARS-CoV-2 spike protein while achieving isotype detection [59]. By producing sensors pre-functionalized with capture ligands, their approach demonstrates how simplification can reduce costs and quicken the time-to-result for BLI diagnostics. Additionally, this assay was used on a BLI instrument compatible with 96-well plates, offering markedly greater throughput.

Further, single-particle interferometric resonance imaging sensors (IRISs) utilize sensor chips arrayed with viral capture probes to count and size viral particles by capturing light transmitted from an LED imaging system [60]. This approach has proven versatile in viral detection, with studies utilizing SP-IRIS to detect VSV [61], H1N1 [62], and filamentous ebolaviruses [60]. In terms of accessibility, this method simplifies the diagnostic process by eliminating the purification steps and reducing sample volumes. Recent improvements in this methodology have further improved its operational simplicity by utilizing multi-wavelength sensing, eliminating the need to capture and process Z-stacked images [63]. Minimizing the need for trained personnel, this instrument could outcompete ELISA methods in detecting the monkeypox virus (MPXV) in 20 min. Doing so while fitting on a benchtop, the improvements in simplicity and time-to-result make this instrument well-suited for clinical use.

The recent developments in BLI have shown how well-fitting this method can be for clinical viral diagnostic applications. The continued iterative improvements in BLI sensing show how the development of novel diagnostic technologies does not end at demonstrating their efficacy but rather how continued refinement helps push proof-of-concept techniques into practical implementation in the clinic. While the quirks of novel biosensors can be worked around more easily in academic labs, where personnel are highly specialized and intimately involved in the development process, honing the technology for simplified use opens the door for untrained personnel to make use of the technology in the field.

### 2.3. Colorimetric and Fluorescent Sensors

Colorimetric sensors represent a class of highly accessible diagnostic tools already commonly used in clinical viral diagnostics. Unlike methods such as PCR, which rely on complex instrumentation and specialized training, colorimetric sensors translate the presence of viral biomarkers, such as antibodies, proteins, or nucleic acids, into visible color changes. This readout mechanism pairs target-specific capture motifs like antibodies or oligonucleotides with chemical or nanomaterial-based signal amplifiers, such as gold nanoparticle aggregation or enzymatic chromogenic reactions (Figure 2B). The resulting signals are detectable by the naked eye or with basic spectrophotometric devices. They represent a rapid, affordable, simple-to-administer testing strategy that retains strong accuracy. These tests are relatively simple and conducive to widespread field testing, evidenced by the widespread adoption of lateral flow assays (LFAs) [64] (Figure 2C). Unlike the more complex optical or electrochemical sensing methods, colorimetric sensors have already established a strong presence in clinical and at-home testing.

In contrast, fluorescent systems can offer improved sensitivity and quantitative abilities by coupling biomarker capture with a light-emissive signal. However, this can come with a reduction in simplicity compared to a simple colorimetric lateral flow assay, as instrumentation would be needed for precise quantification. Current clinical advancements in colorimetric and fluorescent platforms focus on refining sensitivity and usability, with CRISPR-Cas systems emerging as an approach for extremely accurate yet simple diagnostic systems. CRISPR-Cas systems’ strong specificity and programmability enable significantly more sensitive sensing and the ability to tune existing diagnostics for new viruses and mutated variants.

The specificity of CRISPR-Cas-based colorimetric reporter systems has been utilized to perform highly multiplexed diagnostics for multiple viruses and subtypes, simplifying the administration of multi-virus testing by requiring only one substrate [65]. Further, this strong specificity can help reduce the number of sample workup and purification steps by enabling consistent viral sensing directly in serum [66]. With the time sensitivity accompanying viral infections, improvements in the administration and time-to-result of tests make them much more conducive for rapid field testing. Beyond CRISPR systems, other novel colorimetric reporting systems have also been demonstrated. Weerathunge et al. demonstrated a Murine Norovirus (MNV) colorimetric sensor, which utilized a gold ‘nanozyme’ that produces a blue color when the virus displaces color-suppressing aptamers [67]. Offering speed, sensitivity, and simplicity in administration, this example shows how alternatives to established capture and readout mechanisms in colorimetric tests can improve upon an already accessible diagnostic approach.

Despite their simplicity, colorimetric sensors have still exemplified how further refinements can help simplify the interpretation of assay results. Visual readouts are well-suited to the simple, binary detection of an infection, but granularity can be added to colorimetric readouts by coupling computational analysis with the assay. For instance, in a plasmonic nanoparticle-based LFA for SARS-CoV-2 detection, a bimodal detection approach was used to provide an initial visual readout followed by a quantifiable fluorometric reporter [68]. Two-step readouts like this could benefit clinical testing since the first reporter can enable quick responses like isolation and contact tracing. In contrast, the more accurate secondary readout can help clinicians plan treatment regimens. Quantifiable colorimetric readouts would previously require dedicated detection hardware, but recent work has aimed to democratize this process by using hardware that clinics will already have. Since the requirements for processing a colorimetric readout are essentially a light source, a camera, and a means of processing a signal, smartphones and computers can replace a dedicated detector, using their screens as a light source and their built-in cameras for detection [69]. For example, Kim et al. demonstrated a SARS-CoV-2 CRISPR-Cas12 trans-cleavage assay using a smartphone app to quantify the colorimetric readouts of N-protein detection [70]. Using a common dye for the reporting system, the assay was both cheap to produce and able to be run by anyone with a smartphone. Incorporating easy-to-use interfaces in such readout programs makes an additional dimension of viral diagnosis accessible to clinicians and patients without any modification to assay administration. Such assays could also be implemented using commodity hardware in clinics with a low financial barrier.

Colorimetric diagnostics’ simplicity, mainly when administered in a lateral flow assay, has already achieved the accessibility that other diagnostics seek to attain. However, there is always room for refinement. The binary nature of colorimetric readouts leaves much to be desired regarding quantification, and shifts towards more granular readouts offer improvements in this area. Further, the development of multiplexed tests can improve the number of viruses being screened within a single assay, improving the efficiency of each assay.

### 2.4. Non-Optical Methods

While both optical and colorimetric biosensing technologies have seen significant improvements, which improve their clinical viability, electrochemical sensing methods have presented themselves as another viral diagnostic approach (Figure 2D that has also seen recent democratization efforts. These methods, including impedance spectroscopy, voltammetry, and amperometry, offer sensitive and quantitative diagnostic readouts packaged into small, cheap, and simple systems. Impedance spectroscopy monitors the changes in electrical resistance resulting from the binding of an analyte, such as a viral antigen, to a capturing surface. Voltammetry measures binding by analyzing current as a function of applied potential, which can enable viral detection directly through analyte oxidation or reduction and redox-active labels. Voltammetric sensors boast strong sensitivity, rapid measurement, and relative portability, and their sensitivity can be maximized by incorporating specific capture elements like antibodies [71]. Amperometry biosensors achieve detection by measuring current at a fixed potential, where analyte binding results in a proportional, measurable redox reaction at the electrode. The simplicity of amperometric biosensors makes them well-suited to compact and portable biosensing devices, such as those that could be used in clinical and field testing [72]. Nonetheless, there are still practical barriers to seeing electrochemical viral biosensing developments through to the stage of full clinical implementation. Often, electrochemical sensors require a means of additional amplification to improve their sensitivity. Further, surface biofouling becomes particularly prevalent when sensing complex media, as the absorption of proximal proteins, nucleic acids, and lipids to the sensor surface can reduce the long-term stability of the sensing system [73]. Several recent advancements in electrochemical viral biosensors have been made in their simplicity, ease of use, and robustness, bringing them closer to feasible implementation in the clinic.

Alongside the accessibility and cost improvements in optical biosensing, electrochemical viral sensors have also trended towards cheaper and more robust sensing surfaces. These improvements can be tailored to clinical adoption through reusable or disposable single-use sensing surfaces. It was recently demonstrated that gold leaf electrodes—made of extremely thin gold sheets—can be engineered into electrochemical viral biosensors by immobilizing oligonucleotides conjugated with redox-mediating compounds like methylene blue [74]. Since the electrodes could be assembled with gold leaf sheets, razors, stencils, and simple adhesives, they lack the costly cleanroom equipment and labor often required to manufacture gold electrodes. Further, incorporating isothermal amplification into the assay protocol enabled the detection of HIV and HPV without even a thermal cycler. This approach to electrode manufacturing is strongly conducive to mass manufacturing and self-assembly in grassroots testing environments. Advances in electrode materials have also pushed the democratization of electrochemical-based viral diagnostics. Electrodes utilizing graphene have also proven effective for viral biosensing due to their high surface area, conductivity, and compatibility with functionalization. For example, Huang et al. demonstrated a graphene-based Hepatitis B surface antigen sensor, which also touted simple, cleanroom-free manufacturing, that could detect the antigen in human serum [75]. Although the electrode was primarily described as an alternative for lab-based testing, this sensor’s simple manufacturing and disposability could also make it well suited to the clinic as a cheap, pre-manufactured product. The benefits of graphene-based electrodes have been demonstrated more widely in the field, with graphene being incorporated into nanocomposite inks [76] and even bacterially derived cellulose [77]. Incorporating recognition elements into matrices like cellulose and inks enables deposition through screen printing for cheaper and simpler electrode fabrication as an alternative to simple metal electrodes. Screen printing enables the mass production of graphene electrodes, as their surfaces can be engineered to immobilize virus-recognizing moieties like antibodies [78]. While antibody-immobilized electrodes are less affordable and robust than capture moieties like nucleic acids, their specificity is strongly conducive to directly testing patient samples in media such as saliva or serum. Further, the automated nature of printing heads could be combined with multiple inks containing different capture elements to give way to a scalable means of producing multiplexed-capable electrochemical sensors against multiple viral targets.

Electrode technology is one area where electrochemical sensing has advanced, with significant progress in improving sensing hardware for clinical adoption. While academic researchers may find traditional electrochemical setups straightforward to assemble, clinical labs require hardware tailored to their needs—prioritizing speed, throughput, and ease of use. Compact, user-friendly designs are far more practical in clinical settings, and recent studies have demonstrated such solutions for viral sensing. For example, during the COVID-19 pandemic, multiple compact and affordable sensors were developed using accessible electronics and simple readouts. Salahandish et al. created a multiplexed electrochemical impedance spectroscopy (EIS) sensor with commercially available hardware [76], enabling the binary detection of SARS-CoV-2 and variant-specific identification. Similarly, another study demonstrated an amplification-free immunosensor targeting viral RNA using a portable, smartphone-operated electrochemical workstation [79]. This plug-and-play system required just two copies of SARS-CoV-2 per assay, proving that compact hardware can maintain high sensitivity. Further streamlining clinical viral sensing, wireless data transmission allows at-home testing with real-time results sent directly to clinics. One example used laser-engraved graphene electrodes in a SARS-CoV-2 diagnostic device that wirelessly transmitted multiplexed results [80]. Each sensor detected viral proteins or IgG/IgM antibodies, enabling high-sensitivity, multi-target testing. Importantly, these sensing surfaces are also disposable, making them promising candidates for pre-packaged and sterile single-use assays. Such devices could enhance telemedicine by bridging the gap between home and clinic. Moreover, integrating wireless sensors into IoT ecosystems and population monitoring platforms could significantly improve large-scale infection surveillance.

Electrochemical viral biosensing has emerged as a versatile and democratized diagnostic tool, combining sensitivity, portability, and cost-effectiveness to bridge the gaps between research and clinical implementation. Advances in impedance spectroscopy, voltammetry, and amperometry have enabled compact, quantitative systems. At the same time, innovations in electrode materials, such as affordable gold leaf and graphene surfaces, have simplified manufacturing and improved accessibility. Coupled with user-friendly hardware designs, including smartphone-compatible workstations and wireless telemedicine integration, these systems offer multiplexed, high-sensitivity detection without sacrificing practicality. However, barriers to clinical translation persist. Protein-based capture motifs like antibodies still face shelf-life stability and batch-to-batch variability challenges. At the same time, temperature sensitivity during storage and operation can compromise performance in resource-limited settings. Biofouling in complex matrices like blood or saliva also remains a critical limitation, often requiring surface passivation or frequent recalibration [81].

Additionally, many systems still rely on amplification steps or redox labels to achieve clinically relevant sensitivities, adding complexity to workflows. Despite these hurdles, disposable sensing surfaces and scalable production methods (like screen-printed graphene electrodes [82]) underscore the potential for widespread adoption. As electrochemical sensors continue to evolve—addressing stability, standardization, and environmental robustness—their integration into Internet of Things (IoT) ecosystems and clinical workflows shows promise in transforming both point-of-care diagnostics and population-scale surveillance, bringing precision medicine closer to patients and clinicians.

### 2.5. QCM

Gravimetric sensing provides another potential avenue for the clinical detection of viruses, offering operational simplicity by not requiring labeling steps, which may be present in optical sensing methods. Quartz crystal microbalance (QCM) instruments utilize the piezoelectric effect, passing an electric potential through a transducer surface to induce a vibrational oscillation through the contraction and expansion of the quartz crystal lattice [83]. The addition of mass to the surface can be tracked through changes in the transducer’s resonant frequency [84]. If a viscoelastic material is added to the surface, like biological materials, then the dissipation of the oscillations can also be tracked [83]. Like other surface-based sensing methods, QCM can be adapted for viral diagnostics by immobilizing viral capture ligands to the surface. While QCM has generally seen less use in clinical diagnostics, it presents a promising alternative to traditional sensing methods. This is mainly due to the instruments’ low cost, compact size, and operational simplicity.

Like other surface-based methods such as SPR and BLI, it is possible to immobilize capture ligands, such as antigens and antibodies, on the surface for viral detection. The antigen-capture approach has been used for various viruses, including SARS-CoV-2 [85] and African swine fever (ASF) [84]. Antibodies have also been used for viral antigen capture, with studies using this QCM approach to detect HIV-1 [86], bovine leukemia virus [87], and influenza-A [88]. While these approaches were practical, they are not directly conducive to clinical use. The immobilization of proteins or antibodies for capture would need to be performed in the clinic, adding costly and fragile reagents, more complicated assay administration, and more opportunities for procedural error. An alternative approach is likely required to bring QCM into the clinic.

Molecularly imprinted polymers (MIPs) are a potential alternative to protein-based capture on QCM systems. They offer the ability to cheaply functionalize surfaces without the complications of temperature stability that protein capture ligands incur. This means that surfaces can be premade and sent to clinics without storage concerns, and that sensor surfaces would be ready to accept diagnostic samples with no additional workup. MIP surfaces are often washable as well, which makes these surfaces more affordable for clinics by lowering the cost per run [11]. However, molecularly imprinted polymer QCM sensors are not a recent approach in viral diagnostics. For instance, in 2012, Jenik et al. reported the development of an assay to bind different picornaviruses, such as human rhinovirus (HRV) and foot-and-mouth disease virus (FMDV), using stamp imprinting with template viruses [89]. Being able to differentiate between different viral serotypes with signals multiple orders of magnitude stronger than non-specific binding, the process shows how a multi-virus sensor could be built on a QCM platform using MIPs for capture. In 2005, Tai et al. demonstrated a similar molecularly imprinted polymer process, demonstrating that smaller peptides from the linear epitope of the Dengue virus NS1 protein could also be used for detecting viral samples [90]. They achieved the quantitative detection of the NS1 protein in crude and purified samples. Despite these studies not being relatively recent, we have yet to see a mass-produced, readily available MIP solution for clinical adoption. For this QCM approach to reach the clinic, extra development work will need to be performed to bridge this gap.

Generally, QCM presents itself as a promising potential technique for clinical biosensing. It offers high sensitivity and real-time sensing while maintaining relatively simple operation. Although standard QCM can run into limitations when non-rigid analytes are added to the surface, this can be addressed through QCM-D sensing. QCM, like other methods such as SPR, BLI, and electrochemical processes, requires a capture ligand for viral sensing. However, the ability to use more straightforward, more durable capture methods like molecularly imprinted polymers can improve accessibility by simplifying the viral sensor system and by reducing the number of steps to be performed in the clinic itself. While multi-region QCM sensors exist [91], the more affordable instrumentation will likely be limited to individual channels, limiting its throughput.

## 3. Comparing Approaches

Optical biosensing methods carry several advantages that could make them well-suited for clinical use. They are often extremely sensitive, which is beneficial for detecting low viral loads in the early stages of infection. Further, many optical methods like SPR and BLI do not rely on labeling for detection, which reduces the complexity of assay preparation and the possible interfering effects that labels may have on the native interaction being measured for detection. The real-time capabilities of optical methods are a significant strength, enabling the rapid detection of relatively transient interactions. When combined with high-throughput and multiplexed detection strategies, optical biosensors hold the potential for high-throughput mass testing. This does not mean that optical detection is a silver bullet approach to viral detection, though. Often, optical instruments can be costly, and the bulky nature of many optical instruments can make them challenging to deploy in small clinics and for field testing.

A key weakness of many optical instruments is their cost. Both in terms of their consumables—like SPR chips, single-use BLI probes—and the instrumentation itself, their significantly higher prices can make clinical adoption much more difficult. Particularly in resource-limited regions, this factor alone could be enough to eliminate these instruments from contention. In this regard, movements towards affordable instrumentation could help bring these highly sensitive instruments into the hands of more clinics. A barrier to clinical adoption beyond cost is the additional considerations that must be taken into account when using optical instrumentation, such as the effects of temperature, buffer mismatches, and pH. While these factors can be accounted for in a research setting, they add further complexity and training for clinicians. Assay interpretation and false positives can also be a risk for the point-of-care deployment of these instruments, since non-specific binding could be interpreted as a detected infection in untrained hands. Conversely, colorimetric sensors offer far greater simplicity regarding administration and result interpretation but sacrifice some sensitivity and granularity.

Similarly, non-optical methods also have several benefits and drawbacks. Often, instruments like electrochemical sensors are more accessible to clinics than their optical counterparts due to their relatively low cost and size. Their compact nature makes them more feasible for various situations, from smaller clinics to field testing, providing strength in their suitability for both lab and field work. This is further bolstered by advancements in manufacturing capabilities, like printable electrodes and pre-functionalized surfaces, which can provide out-of-the-box functionality for rapid test administration. Their portability means they can also be scaled with less of a need for multiplexed testing, and the simplicity of electrochemical sensors means they could be integrated into portable solutions for community-level testing in the field. While these biosensors are less susceptible to environmental factors like refractive index mismatches, electrochemical sensors can suffer from weakness in their strong susceptibility to biofouling effects, particularly when sensing in complex matrices like serum. This drawback could be improved through prefunctionalizations geared towards surface passivation rather than capture motif immobilization. Further, signal interference can be possible in electrochemical sensors due to other materials in the sample that may be, for instance, redox-reactive. While QCM and electrochemical sensing hardware can be designed with reusability in mind, there is still a cost associated with using the instrumentation, and the degradation of electrodes over time is, to an extent, an unavoidable factor as the electrodes age.

When directly comparing these approaches (Table 1), none are an ideal technique for clinical deployment. While the portability and lower cost of electrochemical sensors may seem appealing, the sensitivity of optical sensors is also important. This means that clinics must weigh the benefits and drawbacks of each diagnostic method, determining which concessions are acceptable while meeting their needs. The innovations we see in recent diagnostic publications—both in optical and non-optical viral biosensing—have aimed to minimize the drawbacks of their respective approaches and minimize the concessions clinics must make. Logistics can also play a significant role in the feasibility of applying novel diagnostics to the point-of-care; as such, the development and production of biosensing surfaces, sensing hardware, and software also significantly determine whether a clinic could feasibly adopt new technologies.

## 4. Diagnostic Development—Hardware and Software

Advancements in viral biosensor technology are essential, but ensuring ease of use and accessibility is crucial for widespread adoption at the point of care. Developing more straightforward instruments and protocols reduces the training required for operators, and lowering the costs of these instruments allows more clinics to utilize state-of-the-art sensing technologies.

Improvements in accessibility for viral diagnostics can be achieved in various ways, including providing pre-made sensing surfaces that offer out-of-the-box functionality, designing instruments that can be easily assembled within the clinic, and creating software that simplifies readouts for healthcare providers and patients. Reducing the number of preparation steps simplifies the administration of diagnostics and minimizes the risk of human error.

Additionally, pre-manufacturing essential diagnostic components, such as microfluidics and functionalized sensor chips, increases reliability and practicality in the field by reducing sample handling and instrumental setup steps for clinic employees. For instance, mass-producing chips with pre-immobilized capture ligands streamlines the setup process, reduces user error, and requires less skilled labor. Simplifying manufacturing processes can also lower costs through economies of scale, making innovative viral diagnostics more accessible.

### 4.1. Three-Dimensional Printing

**Figure 3:**
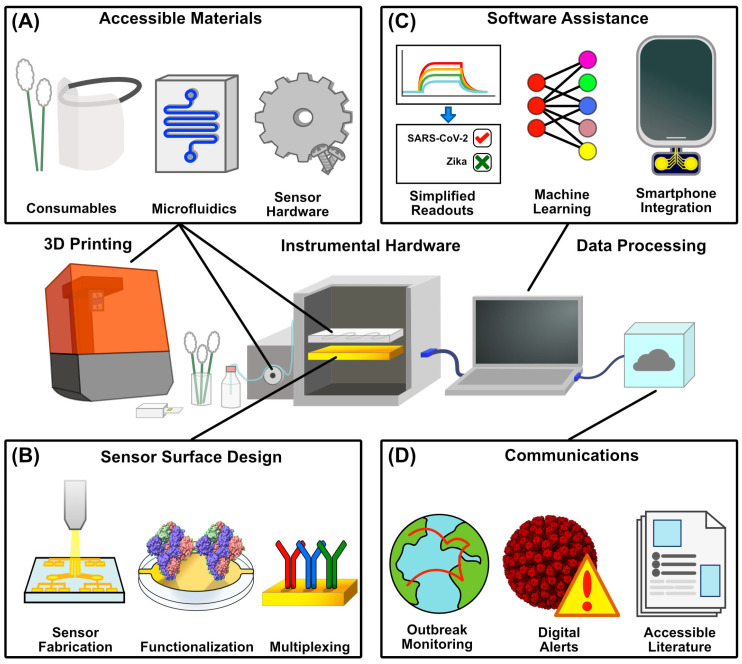
Aspects of POC Accessibility to Novel Diagnostics. (**A**) Additive manufacturing enables the increasingly affordable production of a range of physical diagnostic components, ranging from consumables like swabs and PPE to instrumental hardware like housings and microfluidic systems, as well as replacement internal components for instrumental repair. (**B**) Advancements in sensor surface production can lower the barrier to entry for administering viral assays either through scalable, automated manufacturing, pre-functionalization with immobilized capture motifs, or the utilization of multiple capture motifs for multiplexed sensing (PDB ID 6VXX). (**C**) Software assistance also lowers the barrier for data interpretation through simplified results for complex readouts, the machine learning-assisted processing of increasingly large datasets, and the incorporation of smartphones as processing devices to reduce cost. (**D**) Contact tracing drives outbreak monitoring and digital exposure alerts, easing the burden on clinicians and enabling the preemptive stocking of assay reagents. Additionally, accessible literature can equip both clinicians and the public with a greater understanding of viral infections and the administration of their respective assays (PDB ID 4V7Q)—structures were rendered in UCSF ChimeraX 1.9 [39] and figures were generated in Affinity Designer 2.

Three-dimensional printing has emerged as a pivotal tool in improving the accessibility of diagnostic technology. With the versatility of additive manufacturing and the growing library of 3D-modeled components online, components ranging from instrumental housings and consumables to replacement parts can all be made with commercially, non-clinically oriented hardware (Figure 3A). With the costs of 3D printers falling and the library of open-source designs growing, it is now possible to repair, modify, or even outright assemble instruments by printing parts. In conjunction with the previously discussed improvements in biosensing technology, it is more feasible than ever to build diagnostic systems.

Several studies have demonstrated the ability of 3D printers to be used to make components of diagnostic systems, including microfluidic systems, at high resolutions. Standard methods like extrusion, stereolithography, and material jetting-based 3D printing have demonstrated microfluidic feature resolutions as high as single micrometers, with processes like direct laser writing coupled with photoresists even reaching the nanometer scale [101]. These processes can also be connected with biocompatible materials like polyethylene glycol diacrylate (PEGda) [102] and silicone [103] (among others [104]) to minimize the leaching of material and even enable wearable diagnostic technology. In a more indirect sense, low-cost printers can make molds to cast microfluidics from materials like PDMS [105]. Fluidics are not the only diagnostic components that 3D printing aims to democratize; materials like liquid silica resins and germanate glass have been successfully used to print optical components like prisms and lenses [106], as well as supporting mounts for hardware like smartphones [107] in do-it-yourself (DIY) detectors.

These 3D-printed components have already significantly impacted the realm of viral diagnostics. For instance, aerosol-jet 3D printing was used by Ali et al. to print 3D micropillar arrays that were coated in graphene and functionalized with viral antigens [108]. These 3D-printed sensors were used as electrodes to detect SARS-CoV-2 spike protein and nucleocapsid-specific antibodies in patient sera. Similarly, 3D-printed electrode cassettes were recently developed by Sharma et al., which were combined with paper electrodes to detect dengue and chikungunya antigens in serum through cyclic voltammetry [109]. Krejcova et al. developed a 3D-printable, bead-based microfluidic chip system that isolated paramagnetic beads, followed by voltammetry-based detection [110]. Printed fluidic systems have even been designed to automate ELISA reagent delivery, as demonstrated by Karamzadeh et al., in order to successfully detect the SARS-CoV-2 N-protein in a largely automated format [111]. Extending beyond sensing interfaces themselves, the COVID-19 pandemic saw the widespread use of 3D printing for manufacturing face shields [112], plastic supports for lateral flow assays [113], nasopharyngeal swabs [114], housings for respirator filters [115], and pumps [116]. While some 3D-printable technologies have been directly demonstrated in viral diagnostics, numerous other 3D-printed instruments that could be adapted for detecting viral infections have been developed, demonstrating the continued progress in this field. Examples include microspectroscopy imaging devices [117], PCR thermal cyclers [118,119], photosensitive drug delivery systems [120], quartz crystal microbalances, and electrochemical sensors [121,122].

While the capabilities of 3D printing have been shown across many methods of viral biosensing, continued development will still be needed to lower the barrier of technical knowledge to utilize these tools properly. This is partly aided by the tendency for 3D-printed, open-sourced electronic devices to be made with off-the-shelf components. This aspect is critical in bringing novel diagnostic technology to logistically constrained and underfunded areas. Utilizing generic components helps eliminate the need for niche or proprietary parts, a common hurdle regarding traditional laboratory equipment. Further, the upkeep of these instruments designed with this approach will be far more manageable, as replacement parts and instrument schematics will be more accessible to the end user than what may be seen for commercial biosensing technologies. While publications touting novel sensing methodologies initially seem accessible to clinics, they have little use if the instrumentation required to adopt the methods is prohibitively expensive. Further refinements beyond these initial studies need to be achieved to design easy-to-assemble, printable components to minimize the hardware limitations that clinics may often face.

### 4.2. Sensor Surface Fabrication

Another aspect of bringing more advanced bioanalytical techniques into the clinic for viral biosensing is the ability to produce highly consistent, functionalized, and ready-to-use sensing surfaces. Functionalizing sensing substrates in manufacturing reduces the potential for human error before being sent to a clinic, providing a more ‘plug and play’ process in diagnostics. Further, chip robustness and reusability reduce the cost per run, helping clinics digest the cost of expensive analytical techniques. Pre-functionalizations can range from self-assembled monolayers to polymer and nanomaterial coatings to surfaces pre-functionalized with viral capture motifs [123] (Figure 3B). Metallic sensor surfaces, such as those used in SPR, QCM, and electrochemical diagnostics, can be manufactured in bulk in a clean room using physical deposition methods [124]. This extends beyond metallic surfaces, and thermally grown silicon chips have also been used in viral biosensing [62,63]. Specific surface designs like microarrays, wells, and layered materials can be achieved by coupling physical deposition with photolithography [125,126]. These chips can then be connected to capture elements by carefully selecting the surface chemistry. Generic capture methods are also appealing for clinical use since a single capture protocol could be applied for the simple production of a multi-readout sensor. For instance, gold–thiol chemistry can immobilize thiol-modified capture motifs such as antigen-specific nucleic acids [127]. Materials like paper have even been shown to be an affordable diagnostic substrate. For example, freeze-dried, toehold switch-containing paper substrates have been combined with isothermal amplification and colorimetric readouts to detect the Zika virus [128].

In electrochemical sensing, screen printing offers a more accessible alternative to deposition-based electrode fabrication. Generally, screen-printed electrodes are cheaper and more disposable, and their compatibility with bulk production is conducive to scaled manufacturing [129]. Further, they lack the requirement for a high vacuum and a cleanroom environment for manufacturing, eliminating much of the training required for cleanroom manufacturing. They can enable printing on flexible materials like paper and plastic, whereas other sensor surfaces are constrained to glass or metal substrates [130]. Many printable electrode materials, such as conductive inks containing graphene, polymers like PEDOT: PSS, and nanoparticle-based solutions [131,132,133], are available. Screen-printed electrodes have already been used in viral diagnostics, including the detection of Dengue, HBV, HCV, Zika virus and H1N1, and even in variant-specific SARS-CoV-2 detection [78,134]. Due to the production cost of metallic surfaces like electrodes, screen printing presents an affordable, scalable alternative. Further, quickly disposable electrodes develop single-use, ready-to-run sensing surfaces.

While cheap, single-use sensing surfaces represent one approach to lowering the cost of viral diagnostics, while regenerable functionalized surfaces offer another approach to sustainability. The ability to cleanly strip analytes from a surface enables the reuse of sensing surfaces, which lowers the per-run cost of costly sensing surfaces that cannot be improved by cheaper alternatives (such as gold-coated chips in SPR and QCM). This has been shown using molecularly imprinted polymers [135] and surface-exposed NTA groups to capture histidine-tagged proteins [58,136,137]. Because approaches such as these also lack functional proteins for capture, they benefit from avoiding sensitive storage conditions. This holds the potential for cheaper running costs and more straightforward manufacturing scalability, which could further lower costs.

## 5. Software

Coupled with the development of accessible diagnostic hardware, novel software packages have also been developed to streamline the process of viral diagnostics. The overall goal of pushing new technology into the clinic can be aided by software in several ways (Figure 3C). From broad-scale infection reporting and tracking software to smartphone apps coupled with DIY hardware or even machine learning algorithms built to process complex samples, software advancements go hand in hand with improving the adoption of new diagnostic technology. By streamlining result delivery, simplifying wide-scale testing, and automating the processing of diagnostic data, software tools reduce the administrative burden on clinics, making it easier and more cost-effective to incorporate innovative diagnostics into routine care.

Machine learning is drastically changing the scientific world, and while we find ourselves in the early stages of its advancement into clinical settings, its potential can already be seen. Since machine learning and artificial intelligence tools are well-suited to processing enormous amounts of data, information sources like viral sequences, patient symptoms, and epidemiological trends could all be used to achieve higher-level analysis that individual diagnostic assays cannot attain. Often, machine learning tools are associated with the underlying analysis backing diagnostic development, such as the high-throughput screening of diagnostic target pairs [138]. However, recent advancements in machine learning extend beyond academic labs, with the benefits of their computational power now reaching the clinic. For instance, Khan et al. proposed a tunicate swarm machine learning algorithm for efficient data processing and the transmission of biosensor data between patients and clinicians [139]. The development of such data pipelines is vital for active symptom monitoring and testing recommendations, as it allows for the integration of a range of Internet of Things (IoT) sensors, including those for electrocardiograms (ECGs), body temperature, glucose, and electromyography (EMG). Beyond backend data management, machine learning has also been deployed for clinician and patient use. Machine learning tools can be used to track multiple parameters like symptoms, CD4 counts, and adherence to treatment regimens, which could provide clinicians with strong tools to predict infections and follow through with treatments [140]. Further, broad-scale infection predictions can be made through mass surveillance and publicly available information like search engine trends, where increases in searches related to symptoms could help anticipate waves of infections [141]. Such tools could drastically help preemptively stock assay reagents rather than chasing strong demand, as seen in the COVID-19 pandemic.

With the mass implementation of artificial intelligence tools in every realm of science and technology, it is hard not to see its promise in viral diagnostics. Particularly following the COVID-19 pandemic, we have seen the strong need for infrastructure that can handle diagnostics en masse. The mass data processing capabilities and ease of use of machine learning tools present a novel intersection between accessibility and technical strength, which could show substantial benefits in viral diagnostics. Nonetheless, there still needs to be a gradual, intentional implementation of such tools in clinical testing to ensure their proper use and an understanding of how these tools operate. The tendency for AI tools to ‘hallucinate,’ confidently giving incorrect results, carries much more dire consequences when lives are at stake [142]. In this regard, machine learning tools must be implemented cautiously to ensure that their convenience does not enable blind trust in the readouts given by the diagnostics they may use.

## 6. Smartphone Apps

Mainly due to isolation protocols from the COVID-19 pandemic, the coupling of viral diagnostics and smartphone apps became highly prevalent. Depending on the app, their purpose can range from symptom monitoring to contact tracing, or they can even be used in a smartphone in a home-built diagnostic device. Many apps that ask users to answer symptom questionnaires regularly and alert patients and healthcare providers when symptoms warrant testing have been published [143,144]. The pandemic also saw telemedicine’s widespread adoption and expansion, which gave patients and clinicians a more convenient option [145]. Such types of app can also be coupled with methods to improve diagnostic throughput—like sample barcoding and automated alert systems—to rapidly diagnose and alert patients to positive results without additional visits to the clinic [146,147]. Alongside symptom-monitoring apps, the pandemic also saw the broad adoption of contact-tracing apps, which use the proximity monitoring of other signed-up users to anonymously alert potentially exposed individuals. These apps were mainly popularized due to their adoption by both Apple and Google, as well as their adoption by state-sponsored solutions like TraceTogether and COVIDSafe (from Singapore and Australia, respectively) [146]. Other apps that use smartphones as key components of assays have been developed, serving as detectors. For instance, smartphones have been used in bioluminescent RT-LAMP to detect the Zika virus in urine and saliva and HIV in blood [25]. Similarly, numerous colorimetric COVID-19 tests use smartphones for test readouts in colorimetric and fluorescent tests, taking advantage of the high-quality cameras present in modern smartphones [49,148,149]. Smartphone apps add further improve the usability of modern diagnostics by reducing our reliance on proprietary or complex systems for diagnostic readouts. Given the commonality of smartphones today, a well-designed smartphone app can eliminate the requirement for a clinician to process a readout, leaving them freer to run the assays themselves.

Pre-built diagnostic solutions appeal to clinics due to the simplicity of their setup and learning processes [12,150,151]. Still, the increase in home-built alternatives has also presented a more grassroots approach. Publicly available hardware and software have proven that there are significant cost and logistical benefits associated with the end user taking the burden of assembly upon themselves [152,153,154]. With the barrier to entry for processes like 3D printing continuing to become more accessible, it is becoming far more feasible to take this approach to bring new diagnostic technologies into the clinic [155]. An ideal home-built clinical solution for viral diagnostics could incorporate every aspect of these recent democratization efforts, coupling freely available hardware to open-sourced software packages and using regenerable sensing surfaces to maximize the number of assays per purchase.

## 7. Democratizing Scientific Communications

When discussing advancements that enable clinics to adopt new diagnostic technologies, it is also essential to acknowledge the role of accessible and understandable scientific information, including the pivotal role of scientific journals in disseminating information to clinicians (Figure 3D). In the same way that new technologies have accessibility concerns, particularly in terms of cost and training requirements, there is often a perceived gap between the academic community and the general public. This disparity is mainly present in the sciences that drive diagnostic development, like chemistry, material sciences, and informatics [156]. In recent years, and particularly following the broad impact of the COVID-19 pandemic on the public, many journals have made significant moves to improve the accessibility of their publications. New pilot programs have been adopted, such as translating abstracts from English to other languages like Japanese, Danish, Thai, and Arabic [157]. Recent years have also seen movements that promote disability accessibility in articles. Font sizes, acronym use, and design choices like color and contrast in figures are some considerations that can improve the accessibility of scientific literature for readers [158,159].

Regarding greater public access, journals have increasingly adopted plain-language summaries of their published papers [160,161]. For example, replacing the term ‘hypoxia’ with ‘lack of oxygen’ construes the same idea in a format that a wider audience can understand. There have also been additions like definitions of terms, lists of acronyms, bulleted key points, and video or audio abstracts to accompany paper graphics [162,163,164]. This is a subtle but important aspect in adopting new diagnostics, since accessible scientific literature equips clinicians with the knowledge necessary to administer and understand the diagnostics they may be adopting in their workflows. Improving patient access to scientific literature also helps them understand the procedures they may undergo in a clinic. There is no doubt that in the coming years, artificial intelligence will also see increasing use by clinicians and the public to summarize scientific information. With these tools often being trained by scrubbing vast amounts of data from online sources, it is even more relevant to make scientific details digestible, succinct, and easily accessible to ensure that their responses are grounded in truth.

The push for greater accessibility in scientific publishing mirrors the broader goal of democratizing viral diagnostics. By adopting plain-language summaries, multilingual abstracts, and inclusive design, journals are bridging the gap between academic researchers and clinicians by equipping them with the information necessary to adopt and administer state-of-the-art diagnostic techniques. These efforts also extend to patients whose understanding and receptivity of new diagnostic procedures are enhanced through digestible formats like bulleted key points or audio abstracts. As AI-driven tools increasingly facilitate access to scientific knowledge, ensuring clarity and standardization in publications will grow even more critical to prevent misinformation and align innovation with real-world needs. Accessible literature is supplemental to diagnostic advancement and a foundational step toward equitable adoption across diverse clinical and public health settings.

## 8. Conclusions

The drive to develop faster, more sensitive, and accessible viral diagnostics underscores the clinical world’s commitment to early disease detection. While the COVID-19 pandemic focused on this need, emerging threats like avian flu and monkeypox reinforce the strong demand for innovation. Significant progress has been made in biosensing technologies, enhancing sensitivity, reproducibility, and the detection of novel analytes. However, transitioning from promising research to practical, point-of-care diagnostics still requires overcoming cost, scalability, and ease-of-use challenges. Techniques such as SPR, BLI, and electrochemical sensing offer remarkable sensitivity. However, their adoption in clinical settings hinges on reducing instrument costs, minimizing device footprints, and simplifying operation and data interpretation. In these sensing approaches, democratization efforts have focused on surface pre-functionalizations for ease of use, multiplexing, and physical improvements like cost reduction and a reduction in instrumental footprints. By reducing the cost and barriers to understanding these methods, SPR and BLI present themselves as promising tools for clinical adoption, particularly given their strong sensitivity and label-free sensing capabilities. Non-optical sensing methods have seen similar improvements, with multiplexed sensors and open-source sensing hardware offering affordability benefits in both assays and in the sensing hardware itself. The ability to mass manufacture relatively small electrochemical biosensors makes them a promising option for field testing, particularly regarding their clinical adoption in resource-limited and geographically constrained regions. This is further aided by robust capture motifs like nucleic acids and MIPs, since their stability strongly favors pre-fabrication, shipping, and storage. While these approaches also suffer from biofouling and hardware degradation over time, their relatively cheap cost can overcome these limitations using disposable sensing hardware.

Three-dimensional printing and automated, scalable manufacturing underlie the improvements in sensor technology by reducing the cost and logistical constraints experienced by disenfranchised clinics, allowing them to access otherwise unattainable sensing hardware. Further, the ability to print consumables and instrumental hardware for repairs strongly boosts clinics’ sustainability. With the growing library of open-source hardware, many home-built biosensors are being developed and iterated upon. Self-assembled hardware and democratized biosensing approaches also benefit from a strong software backing. Smartphone-based analysis and simple-to-understand diagnostic readouts lower the training requirement for clinicians while making diagnoses easier for patients to understand. With underlying frameworks for contact tracing, infection reporting, and symptom monitoring, POC clinics can be given strong tools for anticipating and containing wide-scale infection events.

Finally, scientific research reporting works in tandem with innovations in sensing methodologies, hardware, and software provides the public with the information necessary to comprehend the results of diagnostic assays and the phenomena underlying the infections being diagnosed. Initiatives like multilingual plain-language summaries and AI-assisted knowledge translation ensure that advancements reach beyond academic circles to frontline healthcare workers and policymakers. In effect, the democratization of viral diagnostics does not solely lie in the sciences developing the assays. It requires a multidisciplinary effort between scientists, engineers, and journals to not only create new sensing technologies but to put them in clinicians’ hands and equip them with the knowledge needed to utilize them effectively. The future of diagnostics lies not just in detecting pathogens, but in building ecosystems where technology, education, and accessibility converge to empower every community, regardless of resources, to act swiftly against emerging viral threats.

## Figures and Tables

**Table 1:** Comparative analysis of viral biosensing methods. (++++ = Excellent, +++ = Favorable, ++ = Moderate, + = Unfavorable).

Method	Sensitivity	Cost	Complexity	Portability	Clinical/POC Applicability	References
Lateral Flow Assay	+	++++	++++	++++	++++	[92,93]
RT-PCR	+++	+	+	+	++++	[92,93]
ELISA	++	++	++	+	++++	[92,93]
RT-LAMP	++	+++	+++	+++	+++	[93,94,95]
NGS	++++	+	+	+	+++	[96]
Plasmonic/SPR	++++	+	+	+	++	[97,98]
BLI	+++	++	+	+	++	[59,99]
QCM	++	++	++	+++	+	[100]
Electrochemical	++	++	++	+++	++	[74,77,98]

## Data Availability

No new data were created or analyzed in this study. Data sharing is not applicable to this article.
